# The impact of medical and dental education on student antimicrobial resistance literacy in Croatia

**DOI:** 10.1093/jacamr/dlag183

**Published:** 2026-08-31

**Authors:** Caroline Othilie Dedekam Pederssen, Son Lam Pham, Paula Žugec, Jan Homolak, Ivana Šutej, Roger Junges

**Affiliations:** Institute of Oral Biology, Faculty of Dentistry, University of Oslo, Oslo, Norway; Institute of Oral Biology, Faculty of Dentistry, University of Oslo, Oslo, Norway; School of Dental Medicine, University of Zagreb, Zagreb, Croatia; Department of Pharmacology, School of Medicine, University of Zagreb, Zagreb, Croatia; Department of Pharmacology, School of Dental Medicine, University of Zagreb, Zagreb, Croatia; Institute of Oral Biology, Faculty of Dentistry, University of Oslo, Oslo, Norway

## Abstract

**Background and objectives:**

Antimicrobial resistance is a pressing public health concern in Croatia, where antibiotic consumption exceeds the European average. Assessing the impact of education on antimicrobial resistance literacy among future prescribers is a priority, yet parallel evidence from both medicine and dentistry remains limited. Here we evaluated the effect of curriculum-based education on antimicrobial resistance literacy among Croatian medical and dental students across all study years.

**Methods:**

We used a cross-sectional survey to assess dental and medical students’ attitudes towards antibiotics and antimicrobial resistance. Domains included perceptions, knowledge, confidence to prescribe and educational interest. Descriptive statistics were used coupled with statistical testing for comparison across study years.

**Results:**

A total of 692 responses were collected. Antimicrobial resistance was selected as one of the top global priorities by both medical and dental students. While AMR was broadly recognized as a global challenge, significant knowledge gains across study years were more pronounced in dental than in medical students, particularly from year 1 to subsequent years. Over 58% of final-year medical students reported feeling patient-imposed pressure to prescribe antibiotics. For fifth- and sixth-year students, a marked difference in patient communication was observed as >60% of medical students reported discussing antimicrobial resistance with patients very often, compared with only 10% of dental students.

**Conclusions:**

We showed a positive effect of education throughout the study years in both medicine and dentistry, with more evident effects in the latter. High reported pressures to prescribe coupled with a gap in communication practices were observed.

## Introduction

As the future prescribers of antibiotics, education on antimicrobial resistance (AMR) and antimicrobial stewardship (AMS) is a priority in medical and dental education. The teaching of these principles should be either available as dedicated programmes or strongly embedded in courses already present in the curricula, especially in light of growing evidence that knowledge and preparedness deficits persist among healthcare students.^1–8^ Without a strong educational foundation during their formative academic years, these students risk entering clinical practice with insufficient understanding of prescription practices compounded by a lack of awareness of the severity of AMR.^9^ Croatia has a National Action Plan on AMR aligned with the WHO Global Action Plan,^10^ which includes components addressing awareness among healthcare professionals and the general public. Public awareness activities are primarily organized during the World Antimicrobial Awareness Week, complemented by voluntary initiatives led by professional sections within the Croatian Medical Association. However, structured implementation of AMS education specifically targeting medical and dental students remains limited and largely dependent on individual institutional initiative.

Antibiotic usage in Croatia is higher than the average in Europe.^11^ In the community setting, amoxicillin with clavulanic acid has been reported as commonly used as the first choice for treatment,^12^ in spite of available guidelines recommending otherwise.^13^ Such data highlight the need to implement better support systems for prescribers. In Croatian medical schools, AMR is typically addressed within microbiology, pharmacology, and infectious disease modules, with an increasing emphasis on stewardship principles introduced during clinical rotations; however, exposure to formal AMS programmes during these placements is not standardized and largely depends on whether the supervising clinician is personally involved in stewardship activities. In parallel, in dental schools, the inclusion of AMR education is more fragmented, often limited to microbiology courses and teaching within oral medicine or pharmacology units. There is generally less structured exposure to AMS frameworks or guidelines for rational prescribing in dental curricula compared with their medical counterparts. Within this context, the present study aimed to assess the impact of curriculum-embedded educational interventions on AMR literacy among Croatian medical and dental students across all years of study. In addition, for students in the final years of education, we sought to evaluate their confidence to prescribe, frequency of patient communication and the interest in further education on AMR-related topics.

## Methods

Medical and dental students from the University of Zagreb in addition to dental students from the University of Rijeka and the University of Split were invited to participate in this anonymous survey. This study complied with the Declaration of Helsinki and was approved by the Ethics Review Board during March 2023 (05-PA-30-15-2/2023). Data were collected digitally in the period of spring and summer 2023. A previously used instrument developed by our group^1,4^ aiming to assess awareness and knowledge on AMR was used cross-sectionally. Further details on the questionnaire structure and construction are available in the Supplementary Data. Data were curated and analysed in IBM SPSS v.31.0 (Armonk, NY, USA). For visualization, Excel from the Microsoft Office package (Redmond, WA, USA) and GraphPad Prism v.10 (Boston, MA, USA) were used. Averages were reported with the standard error of the mean (SEM). Global challenges and target areas for AMR mitigation were rated by participants on a numerical scale from 1 (not at all important) to 10 (extremely important). Rankings presented in the results reflect mean scores across respondents, without the application of any additional weighting or scoring system. For subsets of analyses, students were divided into three groups according to their year of education in relation to teaching of antimicrobial resistance: (i) first-year students with little emphasis on the topic of AMR, (ii) second- to fourth-year students, for whom teaching is predominantly theoretical and (iii) fifth- to sixth-year students where higher emphasis on clinical teaching is prioritized. For non-parametric data, the Mann–Whitney test was used for comparison of two groups whereas Kruskal–Wallis followed by Dunn’s *post hoc* test was used for assessment of three or more groups. The significance level was set at 0.05.

## Results

The study included a total of 692 participants composed of 268 (38.7%) dental and 424 (61.3%) medical students. General data characteristics are presented in Table S1 (available as Supplementary data at *JAC-AMR* Online). The sample was mostly composed of females (75.3% overall), particularly among dental students (86.9% versus 67.9%), reflecting the actual sex distribution of enrolled students at the participating faculties. The number of participants varied across years of study. The uneven distribution of participants across study years most probably reflects variability in personal encouragement by individual faculty members beyond the standardized email and social media recruitment. Determination of a precise response rate is difficult as the survey was open and anonymous, thus, it was not possible to define the exact number of participants who received the survey. However, >90% of our data were obtained from one university and it is estimated that the response rate in its location was ∼40% for dental students and 25% for medical students across all years, thus yielding a general response rate of ∼32.5%.

**Figure 1.**
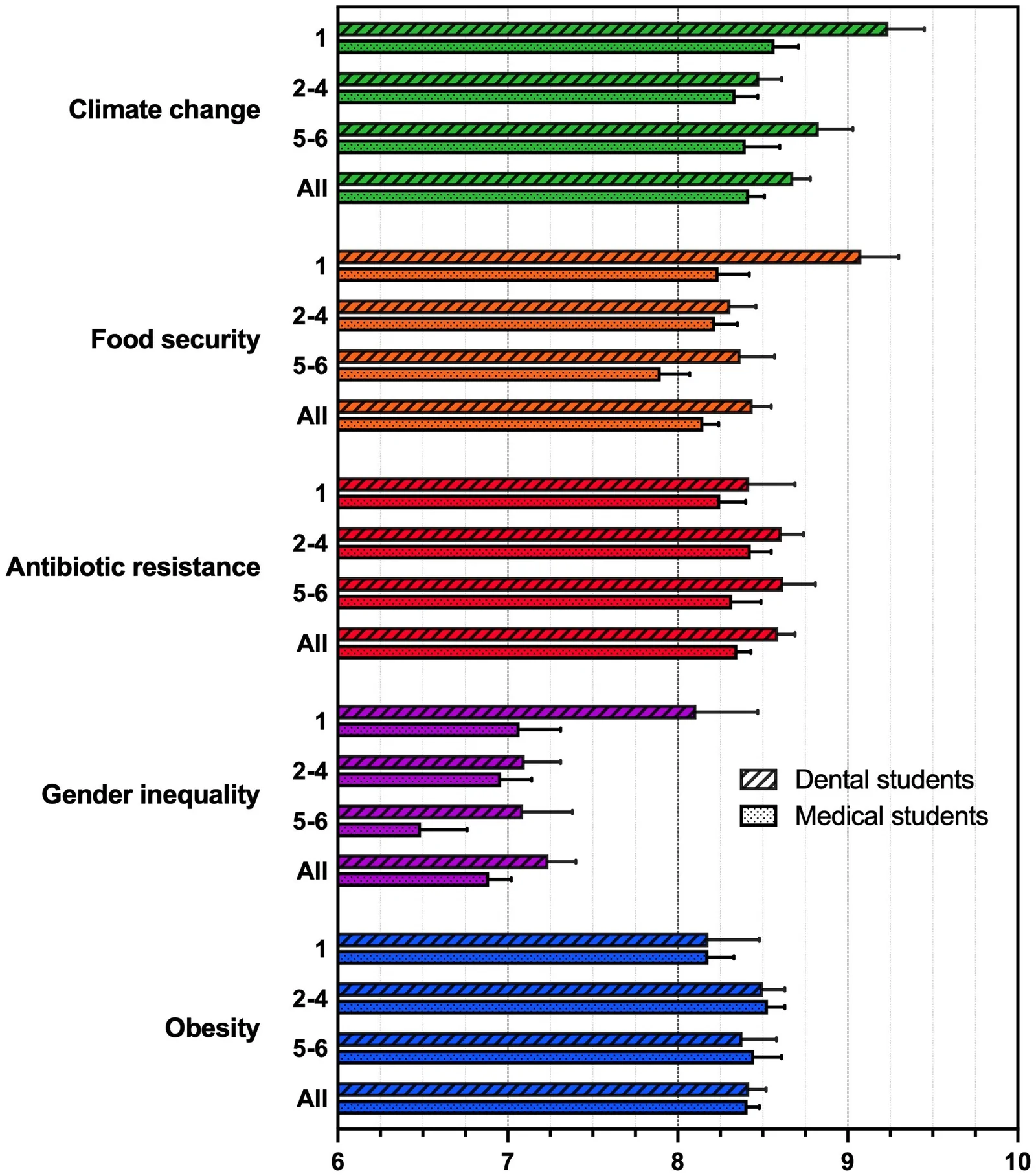
Perceived urgency of dental and medical students regarding five global challenges proposed on a scale from 1 (not at all important) to 10 (extremely important). Bars show average with error bars indicating SEM. The first group of bars in each global challenge represents first-year students, the second group indicates students from the second to fourth year of education, followed by fifth- and sixth-year students. The final set indicates combined responses for all years of education.

**Figure 2.**
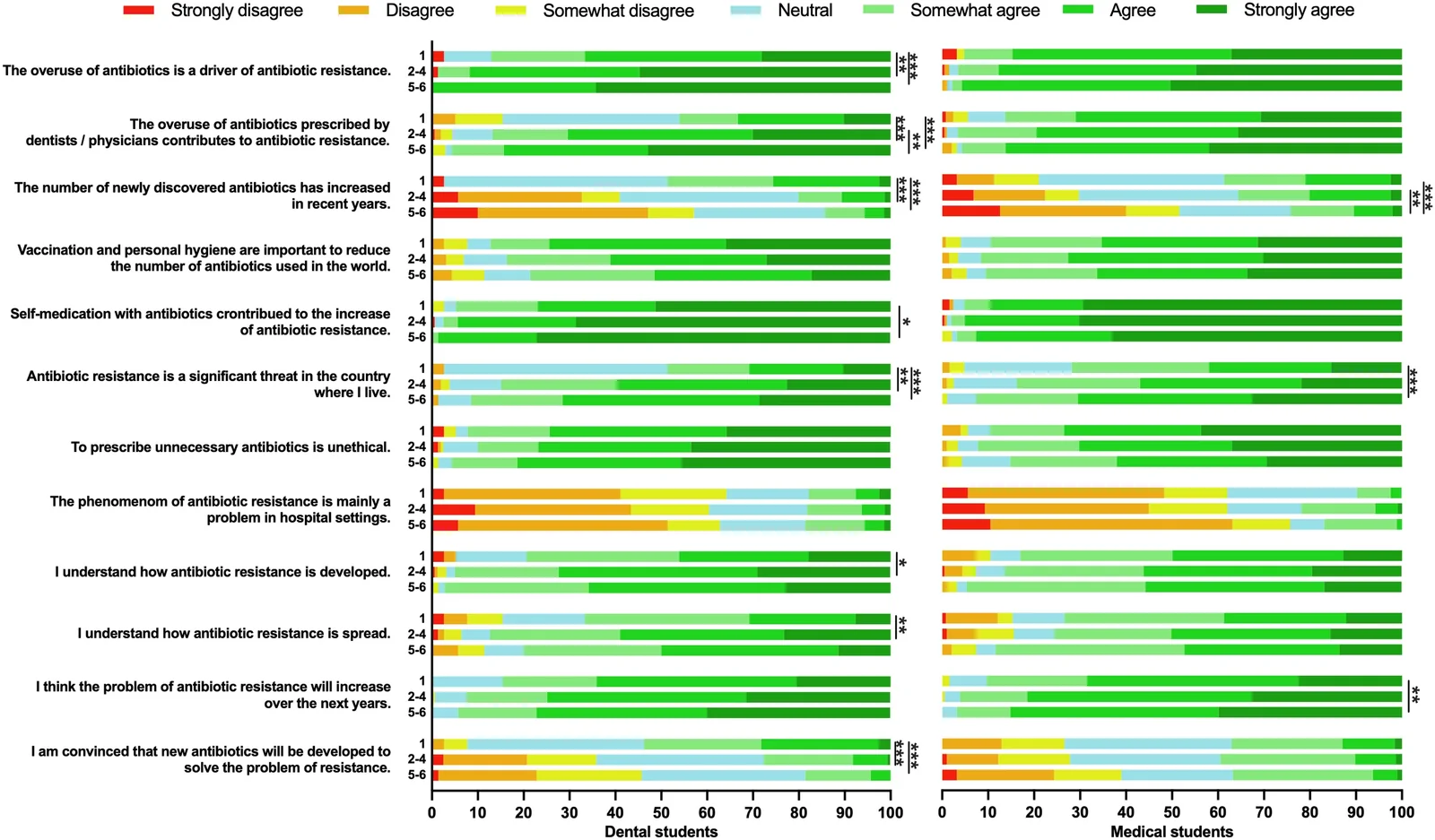
Agreement level of dental and medical students regarding 12 statements on antibiotic use and resistance. Stacked bars indicate relative numbers. The first row of bars for each statement represents first-year students, the second row indicates students from the second to fourth year of education, followed by fifth- and sixth-year students. *P < 0.05, **P < 0.01, ***P < 0.001.

Assessment of global challenges was included to contextualize medical and dental students’ prioritization of AMR relative to other major public health concerns, providing attitudinal framing for the knowledge and literacy findings that follow. For all challenges listed, dental students placed a slightly higher urgency than medical students (Figure 1), which was significant for climate change and food security (*P* < 0.05). The two main target areas that should be prioritized when addressing AMR were ranked in the same order between medical and dental students with antibiotic use in humans (9.33 ± 0.06 and 9.55 ± 0.05, respectively, *P* < 0.01) and public awareness campaigns (9.17 ± 0.06 and 9.32 ± 0.08, respectively, *P* < 0.05). Nearly all dental and medical students responded positively to the importance of AMR in their professions. With regard to the involvement of medicine and dentistry in awareness promoting campaigns on the topic, over half of the participants in each group answered either ‘no’ or ‘not sure’ (Figure S1). For fifth- and sixth-year students, <3% and <14% of dental and medical students, respectively, reported to have heard about the term ‘One Health’. Agreement levels with 12 general statements on different aspects of antibiotic resistance were assessed across student groups (Figure 2).

For patient communication and advocacy, the frequency with which fifth- and sixth-year dental and medical students discuss antibiotic resistance with patients showed distinct patterns (Figure S2). Dental students predominantly indicated that they ‘never’ or ‘rarely’ address the topic, with a fraction reporting discussion occurring ‘very often’. By contrast, medical students reported substantially higher engagement. Overall interest in further education on antibiotic resistance and prescribing was high among both fifth- and sixth-year dental and medical students (Table S2). From 1 to 10, confidence to prescribe antibiotics was reported by final-year dental (7.06 ± 0.24, *n* = 32) and medical students (6.26 ± 0.28, *n* = 35). When pressed in time, 28.1% and 44.4% of final-year dental and medical students, respectively, felt pressure to prescribe antibiotics. Moreover, 40.6% of dental and 58.3% of medical students agreed they felt pressure to prescribe imposed by patients during clinical work.

## Discussion

Equipping future prescribers with adequate knowledge and awareness of AMR is widely recognized as a cornerstone of stewardship efforts, yet evidence on the effectiveness of curriculum-embedded training remains limited. Positive effects of the educational programme across study years were identified for both degree programmes assessed here. The largest difference observed was in dentistry from the first year, before receiving any formal education on microbiology or AMR, compared with subsequent years. The knowledge gain was related to the understanding of the severity of AMR locally in Croatia, the involvement of the prescribing professions and the development gap for antibiotics in the past decades. The observed difference in baseline AMR knowledge between first-year medical and dental students is probably multifactorial. It may reflect differences in pre-university science education, broader media exposure to infectious disease topics or earlier informal exposure to AMR messaging in the medical context.^1,4,14,15^

We observed that final-year students in both medicine and dentistry showed moderate to low confidence to prescribe. These results align with previous data from our group assessing confidence of dental students in other countries from the Asia–Pacific region, North America and northern Europe, which ranged from 6 to 7 for most.^1,4^ Previously, medical students in Croatia have also reported lack of preparedness to prescribe, with a marked reduction in confidence from 2015 to 2019 despite the increase of activities related to AMR and AMS teaching.^8^ It is important to note that previous studies have indicated self-assessment accuracy and confidence in medicine students to poorly correspond with levels of knowledge.^16–18^ Patient pressure and lack of time were reported frequently in our study as potential stressors, particularly for medical students. This finding could be attributed to a larger variety of patient care scenarios requiring antibiotic intervention encountered in medical practice versus dental practice. In dentistry in particular, a recent study in the region showed that about two-thirds of Croatian dentists felt they had received adequate training to prescribe during their studies, with the number dropping to less than half of the participants in both Bosnia and Serbia.^19^ A study examining knowledge and prescription patterns among dentists in Croatia, Serbia and Bosnia and Herzegovina highlighted that 22.3% of Croatian dentists prescribed antibiotics at the request of patients, indicating a concerning gap in communication between practitioners and patients.^19^ This issue is further underscored by our survey results, which revealed that >90% of fifth- and sixth-year dental students rarely or never discuss AMR with their patients, in contrast to >60% of medical students who frequently engage in such discussions. Previous studies in the region have also suggested that working in multidisciplinary teams coupled with communication skills are key areas that should be better prioritized regarding AMS competencies in medicine.^8,20^ Further integration of dentistry into interprofessional healthcare is a priority also in view of AMR and AMS.^21^ Overall, the contact between caregiver and patient plays a key role in advocacy towards safe drug use.

Several limitations of this study should be acknowledged. The cross-sectional design precludes causal inference and does not allow tracking of knowledge development within the same cohort over time. The overall response rate was estimated at ∼30% that, while consistent with comparable online survey studies, introduces the risk of non-response bias, particularly among final-year students whose clinical workload may have reduced participation. The grouping of students into three educational stages was based on the general structure of AMR-related teaching in Croatian curricula, but the precise emphasis on AMR topics may vary across institutions and individual courses within these stages. Self-reported measures of confidence and communication frequency are subject to social desirability bias and may not accurately reflect objective competence or actual clinical behaviour. Regarding the strengths, we address a gap in the literature by assessing AMR literacy in students from all years of dental and medical education in Croatia. Furthermore, by using a survey instrument previously validated and used in other world regions, our findings contribute to a Central and Eastern-European perspective to an emerging cross-continental evidence base, a region notably underrepresented in AMR education research despite its relevance to antimicrobial prescription patterns. Building on these findings, future studies should move beyond cross-sectional knowledge assessment towards pre–post intervention designs embedded within curricula, longitudinal cohort studies tracking the same students across years and qualitative approaches exploring perceived barriers to AMR communication and stewardship practice among students.

### Conclusions

This study provides parallel evidence on the impact of curriculum-embedded education on AMR literacy among Croatian medical and dental students. Education exerted a positive effect across both degree programmes, with the most pronounced knowledge gains observed in dental students from year 1 to subsequent years of study. Final-year students in both programmes demonstrated moderate prescribing confidence alongside considerable patient- and time-related pressure to prescribe. A critical gap in One Health awareness was identified across both cohorts. Patient communication regarding AMR differed markedly between the two study programmes, with dental students engaging substantially less frequently than their medical counterparts. Collectively, these findings indicate a strong demand for enhanced AMR and AMS education in both curricula, with particular emphasis on preclinical competencies and communication skills.

## Supplementary Material

dlag183_Supplementary_Data

## Supplementary data

Figures S1 and S2 and Tables S1 and S2 are available as Supplementary data at *JAC-AMR* Online.
