# Supplementary material for: The impact of medical and dental education on student antimicrobial resistance literacy in Croatia
Source: JAC Antimicrob Resist. 2026 Aug 31;8(4):dlag183. doi: 10.1093/jacamr/dlag183 (PMC13526504; doi:10.1093/jacamr/dlag183)
Supplement: Supplementary file 1 [file dlag183_supplementary_data.docx]

**Supplementary material to**

**The impact of medical and dental education on student antimicrobial resistance literacy in Croatia**

Caroline Othilie Dedekam Pederssen^1#^, Son Lam Pham^1#^, Paula Žugec^2^, Jan Homolak^3^, Ivana Šutej^4^, Roger Junges^1^*

^#^both authors contributed equally

^1^ *Institute of Oral Biology, Faculty of Dentistry, University of Oslo, Oslo, Norway*

^2^ *School of Dental Medicine, University of Zagreb, Zagreb, Croatia*

^3^ *Department of Pharmacology, School of Medicine, University of Zagreb, Zagreb, Croatia*

^4^ *Department of Pharmacology, School of Dental Medicine, University of Zagreb, Zagreb, Croatia*

^*^corresponding author

**Questionnaire structure and construction**

A previously employed instrument developed by our group^1, 2^ aiming to assess awareness and knowledge on AMR was utilized. The questionnaire contained three areas of domain: perceptions and knowledge, confidence to prescribe, and perceived needs for further education. The first domain encompassed participants' perceptions of AMR as a global challenge, their level of agreement with statements on antibiotic use and resistance, and their beliefs regarding mitigation strategies. The second and third domains of the instrument encompassed confidence to prescribe and needs for further education. The questions in the first domain are generally broad and do not require profession-specific knowledge. In the second domain, we have omitted specific scenarios where knowledge on the profession is required. Thus, we proceeded with two nearly identical versions of the questionnaire containing only small wording adaptations such as:

*What is your level of agreement with each statement?*

*The overuse of antibiotics prescribed by dentists contributes to antibiotic resistance.*

(version for dental students)

versus

*The overuse of antibiotics prescribed by physicians contributes to antibiotic resistance.*

(version for medical students)

The questionnaire contained mostly close-ended questions and was structured to be answered in 5-10 minutes to avoid fatigue. The instrument was translated into Croatian by native-speaking subject-matter experts and back-translated into English to verify conceptual equivalence and linguistic accuracy. An invitation to participate, including a link to the online survey, was distributed to the study population via email. To maximize response rates, up to three reminder emails were sent, and participation was further encouraged verbally by the research team and through social media channels.

**References**

1.	Ghafoor S, Salvadori G, Kino S et al. Insights Into Antimicrobial Resistance From Dental Students in the Asia-Pacific Region. *Int Dent J* 2025; **75**: 263–72.

2.	Bajalan A, Bui T, Salvadori G et al. Awareness regarding antimicrobial resistance and confidence to prescribe antibiotics in dentistry: a cross-continental student survey. *Antimicrob Resist Infect Control* 2022; **11**: 158.

**Table S1.** Characteristics of dental and medical students participating in the study.

|  |  | Dental students  n (%) | Medical students  n (%) |  | Total  n (%) |
| --- | --- | --- | --- | --- | --- |
| Participants | |  |  |  |  |
|  |  | 268 (38.7) | 424 (61.3) |  | 692 (100) |
| Age |  |  |  |  |  |
|  | Mean (SEM, Median) | 21.98 (±.11, 22) | 21.49 (±.09, 21) |  | 21.68 (±.07, 22) |
| Sex |  |  |  |  |  |
|  | Female | 233 (86.9) | 288 (67.9) |  | 521 (75.3) |
|  | Male | 35 (31.1) | 130 (30.7) |  | 165 (23.8) |
|  | Other | 0 (0) | 6 (1.4) |  | 6 (0.8) |
|  |  |  |  |  |  |
| Research activities | |  |  |  |  |
|  |  | 20 (7.5) | 50 (11.8) |  | 70 (10.1) |
|  |  |  |  |  |  |
| Study year | |  |  |  |  |
|  | First | 39 (14.6) | 124 (29.2) |  | 163 (23.6) |
|  | Second | 35 (13.1) | 57 (13.4) |  | 92 (13.3) |
|  | Third | 60 (22.4) | 110 (25.9) |  | 170 (24.6) |
|  | Fourth | 64 (23.9) | 38 (9) |  | 102 (14.7) |
|  | Fifth | 38 (14.2) | 59 (13.9) |  | 97 (14) |
|  | Sixth | 32 (11.9) | 36 (8.5) |  | 68 (9.8) |

**Table S2.** Interest in fifth- and sixth-year dental and medical students in receiving further education on selected topics with regards to antibiotic resistance and prescription practices.

| **Topic** | **Dental students** | | | | | | **Medical students** | | | | | |
| --- | --- | --- | --- | --- | --- | --- | --- | --- | --- | --- | --- | --- |
|  | **Yes** | | **No** | | **Not sure** | | **Yes** | | **No** | | **Not sure** | |
|  | **n** | **%** | **n** | **%** | **n** | **%** | **n** | **%** | **n** | **%** | **n** | **%** |
| Spread of resistance | 62 | 88.6 | 8 | 11.4 | 0 | 0 | 80 | 84.2 | 10 | 10.5 | 5 | 5.3 |
| Development of resistance | 56 | 80 | 13 | 18.6 | 1 | 1.4 | 72 | 75.8 | 18 | 18.9 | 5 | 5.3 |
| Drug interactions | 59 | 84.3 | 9 | 12.9 | 2 | 2.9 | 81 | 85.3 | 7 | 7.4 | 7 | 7.4 |
| Antibiotic prescription - treatment | 61 | 87.1 | 8 | 11.4 | 1 | 1.4 | 83 | 87.4 | 10 | 10.5 | 2 | 2.1 |
| Antibiotic prescription - prophylaxis | 51 | 72.9 | 15 | 21.4 | 4 | 5.7 | 85 | 89.5 | 8 | 8.4 | 2 | 2.1 |
| Antibiotic resistance in humans, animals, and the environment | 59 | 84.3 | 6 | 8.6 | 5 | 7.1 | 95 | 100 | 0 | 0 | 0 | 0 |

**Fig S1.** Responses from dental and medical students regarding perceptions on AMR. First block shows answers regarding involvement of the profession in national and international campaigns to promote awareness on AMR. Second block shows responses regarding knowledge of the concept of 'One Health'. Data are shown in relative numbers divided by groups.

**Fig S2.** Relative frequency of conversation with patients regarding the topic of antibiotic resistance. **(A)** Fifth- and sixth- year dental students, and **(B)** Fifth- and sixth-year medical students.
